# Ketamine as a potential cognitive enhancer in neurological disorders: evidence from preclinical and clinical studies

**DOI:** 10.3389/fneur.2026.1786249

**Published:** 2026-03-30

**Authors:** Jose E. Leon-Rojas, Guido Mascialino, Lizeth Vinueza Mera, Mario S. Hinojosa-Figueroa, Cristina F. Navas Arias, Estefano D. Cadena Barberis, Amanda Sacks-Zimmerman

**Affiliations:** 1 Cerebro, Emoción y Conducta (CEC) Research Group, School of Medicine, Universidad de Las Américas (UDLA), Quito, Ecuador; 2 Health Sciences Department, Universidad Pública de Navarra, Pamplona, Spain; 3 NeurALL Research Group, Quito, Ecuador; 4 Department of Neurological Surgery, Weill Cornell Medicine, New York, NY, United States; 5 Acheron Psychiatry, New York, NY, United States

**Keywords:** cognitive function, ketamine, neurological disorders, neuroplasticity, traumatic brain injury

## Abstract

Subanesthetic ketamine shows rapid neuroplastic and antidepressant effects in psychiatric conditions, prompting interest in its potential relevance for cognitive dysfunction in neurological disorders. Cognitive deficits are widespread across traumatic brain injury, stroke, epilepsy, and neurodegenerative diseases, yet treatments remain primarily compensatory. Ketamine’s actions on glutamatergic signaling, synaptogenesis, and neuroinflammation suggest possible cognitive implications, but its specific effects on cognition in neurological populations remain unclear. This systematic review evaluated evidence on the effects of ketamine on cognitive functioning in neurological diseases and injuries characterized by cognitive impairment. A systematic search of three databases through February 10, 2024, was conducted following Preferred Reporting Items for Systematic Reviews and Meta-Analysis 2020 guidelines, including animal or human studies administering ketamine or its derivatives with cognitive outcome measures. Twenty-two studies met inclusion criteria: twenty-one animal studies and one human study. In animal models of traumatic brain injury, epilepsy, cerebrovascular disease, Parkinson’s disease, and infectious encephalopathy, a subject-level vote count in rodents indicated positive cognitive effects in 93.2% of subjects, particularly in working memory and spatial learning, while null effects appeared in 4.1% and negative effects in 2.7%. The only human study, conducted in patients with Huntington’s disease, reported short-term, dose-dependent cognitive worsening after escalating intravenous ketamine. Overall, preclinical evidence indicates potential cognitive effects in animal models of neurological injury; however, human evidence remains extremely limited and does not currently support clinical cognitive enhancement. Further controlled clinical studies are needed to clarify safety, mechanisms, and translational relevance in neurological rehabilitation.

## Introduction

Ketamine, an NMDA receptor antagonist originally developed as an anaesthetic agent, has garnered significant interest in recent years due to its therapeutic effects in psychiatric conditions. Most notably, ketamine has been increasingly investigated for the treatment of major depressive disorder, particularly in cases of treatment-resistant depression (TRD), where subanesthetic doses (0.5 mg/kg) have been associated with rapid antidepressant effects (1, 2). In clinical practice, ketamine and its S-enantiomer esketamine are administered at subanesthetic doses under structured clinical supervision due to their psychoactive effects and potential cardiovascular and dissociative responses. Real-world studies and clinical reviews highlight the importance of monitored administration settings and standardized protocols to ensure safety and optimize treatment outcomes in patients with TRD (3, 4).

Beyond these psychiatric applications, emerging evidence suggests that subanesthetic ketamine may exert neuroprotective and neurotrophic effects through the modulation of neuroplasticity-related mechanisms (5). These biological properties have prompted growing interest in the potential neurocognitive implications of ketamine in neurological disorders (6). However, there is a need to establish research on the impact of ketamine on neurocognitive functioning for heterogenous neurological disorders; as currently this relationship has not been fully elucidated.

Neurological disorders such as Parkinson’s disease, multiple sclerosis, epilepsy, and traumatic brain injury are frequently accompanied by cognitive deficits that profoundly affect patients’ daily functioning and quality of life. Existing treatment strategies for these impairments are essentially compensatory, aimed at maximizing function rather than directly improving cognition. Thus, there is a pressing need for innovative therapeutic approaches. Ketamine’s pharmacological profile, which includes the capacity to induce neuroplastic changes, positions it as a potential candidate for ameliorating cognitive dysfunction in these populations (7).

Furthermore, many neurological disorders are often accompanied by depressive states, which can compound effects on cognition including information processing, attention, and higher order cognitive functions such as working memory and flexibility (8). Sequalae of brain dysfunction, whether acquired injuries or neurodegenerative processes, include both neurocognitive and emotional changes. Therefore, a pharmacological agent that provides both an antidepressant as well as neuroplastic effect may exert positive influence on neurocognitive outcomes. The exploration of neurological therapeutic applications is a natural outgrowth of findings of the effect of subanesthetic ketamine on cognitive functioning in psychiatric populations. Gill et al. (9) conducted a systematic review and meta-analysis demonstrating the antidepressant efficacy of racemic ketamine and esketamine in treatment-resistant depression. While these findings primarily relate to mood outcomes, they have prompted further investigation into whether ketamine may exert additional cognitive effects beyond its antidepressant properties. Although ketamine treatment for TRD is typically delivered within controlled clinical settings with monitoring protocols, evidence regarding its direct pro-cognitive effects remains limited and heterogeneous. Interestingly, Gill et al. (9), demonstrated through a systematic review, that low dosages of ketamine improved visual memory and working memory in individuals with TRD suggesting that cognitive improvements may contribute to the acute reduction of depressive symptoms. A clinical study evaluating cognitive performance following subanesthetic ketamine in treatment-resistant depression reported largely preserved cognitive function, with mixed domain-specific findings (10).

Moreover, depressive symptoms are prevalent across many neurological conditions, often compounding cognitive deficits in domains such as information processing, attentional control, working memory, and executive functioning. Given the interplay between affective and cognitive disturbances, a pharmacological agent capable of exerting both antidepressant and neuroplastic effects could potentially enhance neurocognitive outcomes. This dual-action potential of ketamine, already observed in psychiatric populations and supported by clinical safety and tolerability data (11), provides a rationale for investigating its application in neurological rehabilitation.

Despite the growing evidence of pro-cognitive effects in psychiatric populations, there is a paucity of literature on pro-cognitive effects of subanesthetic ketamine in the neurological, brain injured population.

The potential pathways through which ketamine may ameliorate cognitive dysfunction are underexplored; however, both its pro-cognitive effects and antidepressant effects are thought to arise from its multifaceted pharmacological profile. Sub-anaesthetic doses of ketamine can promote synaptogenesis and neuroplasticity in several brain areas, such as the medial prefrontal cortex (mPFC) and some limbic regions (12). More specifically, ketamine’s antagonism of NMDA receptors leads to increased synaptic plasticity, specifically activating prefrontal glutamate transmission which while having an antidepressant effect, can also enhance the functions of the prefrontal cortex, including cognitive flexibility and problem solving (13). Consistent with this concept, ketamine has been shown to enhance excitatory synaptic function via glutamate ion channels within the frontal cortex and hippocampus (14). Ketamine-induced activation of glutamate receptors contributes to release of brain derived neurotropic factor (BNDF) via activation of calcium channels (15). BDNF regulates synaptic efficacy, which may facilitate positive neuroplastic changes, specifically in the areas of memory and learning, within the context of ketamine use (16, 17). Lastly, ketamine also modulates neuroinflammatory processes, reducing microglial activation and inflammatory cytokine release. These actions, combined with potential increases in BDNF levels and neurogenesis, likely contribute to ketamine’s cognitive benefits. Ketamine’s upregulation of BDNF which contributes to synaptogenesis is a central adaptive plasticity mechanism (3). The precise mechanisms underlying ketamine’s pro-cognitive effects require further investigation.

In animal models, ketamine administration has been shown to activate many downstream signals implicated in neuroplasticity (13). Recovery from neurocognitive dysfunction within the context of acquired brain injury depends on neuroplastic changes reflected in the mechanisms of action of ketamine.

From an experiential perspective, the use of subanesthetic ketamine has been described as enhancing flexibility and clarity in thinking, as well as stimulating creativity through facilitating access to subconscious processing (18). Such descriptions of the psychological impact of ketamine may have implications for neurocognitive performance, specifically in the areas of higher order cognitive domains.

Despite the growing interest in the therapeutic potential of ketamine, the evidence regarding its effects on cognitive functioning in neurological diseases remains isolated to the animal literature and inconclusive. There is a dearth of studies examining the impact of ketamine on neurocognitive functioning, specifically in those with acquired brain injury as most studies have examined the impact of ketamine on reduction of symptoms related to mood and behavioral dysfunction. The field of neurorehabilitation has a long-standing history of effective use of multiple techniques and strategies to enhance cognitive functioning subsequent to acquired brain injury. Supplemental pharmacological intervention to enhance efficacy of neurorehabilitation and patient experience of treatment may yield benefits to outcome.

Should ketamine induce a window of neuroplastic changes rendering neurological processes more receptive to the impact of neurorehabilitation, subanaesthetic ketamine could be a beneficial component of the practice of rehabilitation psychology. In order to examine the role of ketamine as a potential intervention in neurological disorders in general, and acquired brain injury, the current systematic review will incorporate available studies in extant literature that have investigated the use and impact of ketamine on various models of brain injury. This review aims to systematically evaluate and synthesize the available evidence on the impact of ketamine on cognitive functioning in various neurological diseases and injuries characterized by cognitive dysfunction. By critically appraising the existing literature, we seek to elucidate the potential benefits and limitations of ketamine as a therapeutic agent for cognitive impairment in neurological diseases. The findings of this review may have important implications for the development of novel treatment strategies aimed at improving cognitive function and quality of life in patients with neurological disorders.

## Materials and methods

Our systematic review was written following the Preferred Reporting Items for Systematic Reviews and Meta-Analysis (PRISMA) 2020 guidelines (19). We followed an appropriate protocol, and no deviations occurred during its implementation. Furthermore, preregistration was attempted at PROSPERO, but it was denied because PROSPERO does not accept registration of systematic reviews that include non-human studies as explicitly stated in its eligibility criteria.^1^

### Eligibility criteria

The inclusion criteria consisted of experimental studies that involved the use of ketamine or its derivatives (r-ketamine, s-ketamine, or racemic ketamine), without adjunctive medications together with a measurement of cognitive outcomes pre- and/or post-administration in animal models or humans with an organic neurological disease; studies in both English and Spanish were also included. We excluded studies that used ketamine in cognitively healthy populations, lacked cognitive outcome measures, assessed perioperative cognitive disorders, or included participants with co-morbid psychiatric diseases. We also actively excluded studies in which ketamine was administered alongside an additional active pharmacological co-intervention that could modify neurobehavioral or cognitive outcomes. Background agents used for model induction, anaesthesia, analgesia, or peri-procedural care were permitted only when applied equally across ketamine and comparator groups and when ketamine remained the primary experimental contrast. Any studies involving an additional active co-intervention that could affect cognitive outcomes were explicitly flagged and excluded.

### Information sources and search strategy

We queried PubMed, Scopus, and the Virtual Health Library (VHL) databases from their inception until February 10, 2024; no filters were applied. The full search strategies for each database are provided verbatim in Supplementary material 1. We used a combination of medical subheadings (MeSH) and search terms that included the following keywords: ketamine, neurocognitive impairment, cognitive dysfunction, organic neurocognitive disease (and specific aetiologies like stroke, trauma, neoplasms, epilepsy, etc), cognition, cognitive function, recovery, rehabilitation, attention, memory, and executive function, among others.

### Data management

All articles were imported and handled in Ryyan (a web-based systematic review software) developed by the Qatar Computing Research Institute^2^ for screening and duplicate resolution; this aided in minimizing bias, data entry errors, and selection errors.

### Selection process

The aforementioned eligibility criteria were used to screen all titles, abstracts, and keywords by two blinded and independent reviewers (in duplicate) using Ryyan. The selection process included the application of two filters. At first, all articles were assessed by reviewing the titles, abstracts, and keywords; the second filter involved a thorough full-text review. Any conflicts or discrepancies were solved by a third reviewer through discussion and mutual consensus.

### Data items and data synthesis

After the selection process, data from the chosen articles was extracted, compiled, and organized in a Microsoft Excel (Microsoft Corporation, Redmond, USA) spreadsheet; the following variables were extracted: author, year of publication, study design, study population (human/ animal), if animal (mice/rats/other), number of test subjects, number of males, number of females, type of ketamine administered, number of doses administered (single/multiple), moment of dosage administration (early <48 h or delayed >48 h), ketamine dosage (in mg/kg), timing of administration (before and/or after injury), type of organic neurological disease, neurocognitive test performed, time of neurocognitive test, ketamine influence in cognition (positive/negative/unchanged), and reported adverse events.

To ensure transparency and reproducibility of the subject-weighted synthesis, we established explicit operational definitions for coding cognitive outcomes as positive, negative, or unchanged. Cognitive effects were classified relative to the disease-model control group receiving vehicle or placebo rather than to healthy controls. Each ketamine-treated group within a study was considered an independent analytical unit and weighted according to the number of subjects in that group.

A “positive” effect was defined as a statistically significant improvement in at least one cognitive domain compared with the disease-model control group, provided no statistically significant worsening was reported in any cognitive domain at the same principal timepoint. A “negative” effect was defined as statistically significant worsening in at least one cognitive domain compared with the disease-model control group. An “unchanged” effect was defined as the absence of statistically significant differences between ketamine-treated and control groups across all reported cognitive domains.

When multiple cognitive tests were reported, we prioritized core domains such as working memory, spatial learning, recognition memory, and executive function; if conflicting findings occurred within the same treatment group and timepoint, we conservatively classified the outcome according to the most adverse statistically significant result. Furthermore, when multiple post-treatment timepoints were presented, we coded the earliest timepoint intended to assess ketamine’s cognitive effect unless the study clearly identified a primary endpoint.

Finally, because ketamine can have acute psychoactive and sedative effects that may confound behavioural testing, we extracted the timing of cognitive assessment relative to dosing (time from last ketamine administration to cognitive testing) whenever reported, alongside route of administration and dosing schedule.

### Bias assessment

We used the National Heart, Lung, and Blood Institute’s (NHLBI) study quality assessment tools^3^ for bias assessment in human studies and SYRCLE’s risk of bias tool for animal studies (20). These tools comprised a set of questionnaires tailored to the specific type of study design being evaluated. The degree of bias for the NHLBI tool was categorized as low, moderate, or high based on the percentage of affirmative responses to the questions. Low risk of bias was indicated if 80% or more of the questions were answered as yes; moderate risk of bias was between 50 and 79% and high risk of bias was less than 50%. For the SYRCLE’s risk of bias tool, a summary risk of bias calculation is not recommended so we provided the answers to each of the domains considered in the tool. Risk of bias was assessed by two blinded reviewers, in duplicate, with a third reviewer for discrepancies.

### Effect measures and synthesis methods

Our study aimed to showcase the potential utility of ketamine administration in cognitive improvement in those with an organic neurological disease through the means of a qualitative systematic review reporting absolute and relative frequencies as our outcome measures. A meta-analysis was not feasible due to the significant heterogeneity between studies and the scarcity of information (i.e., the scientific literature contains multitude of studies considering ketamine for psychiatric disorders while neglecting organic neurological conditions that often present with cognitive impairments). Furthermore, as we wanted to showcase the current state of the literature, we included different types of neurological conditions as well as animal/human studies that are not comparable.

## Results

### Study selection

**Figure 1:**
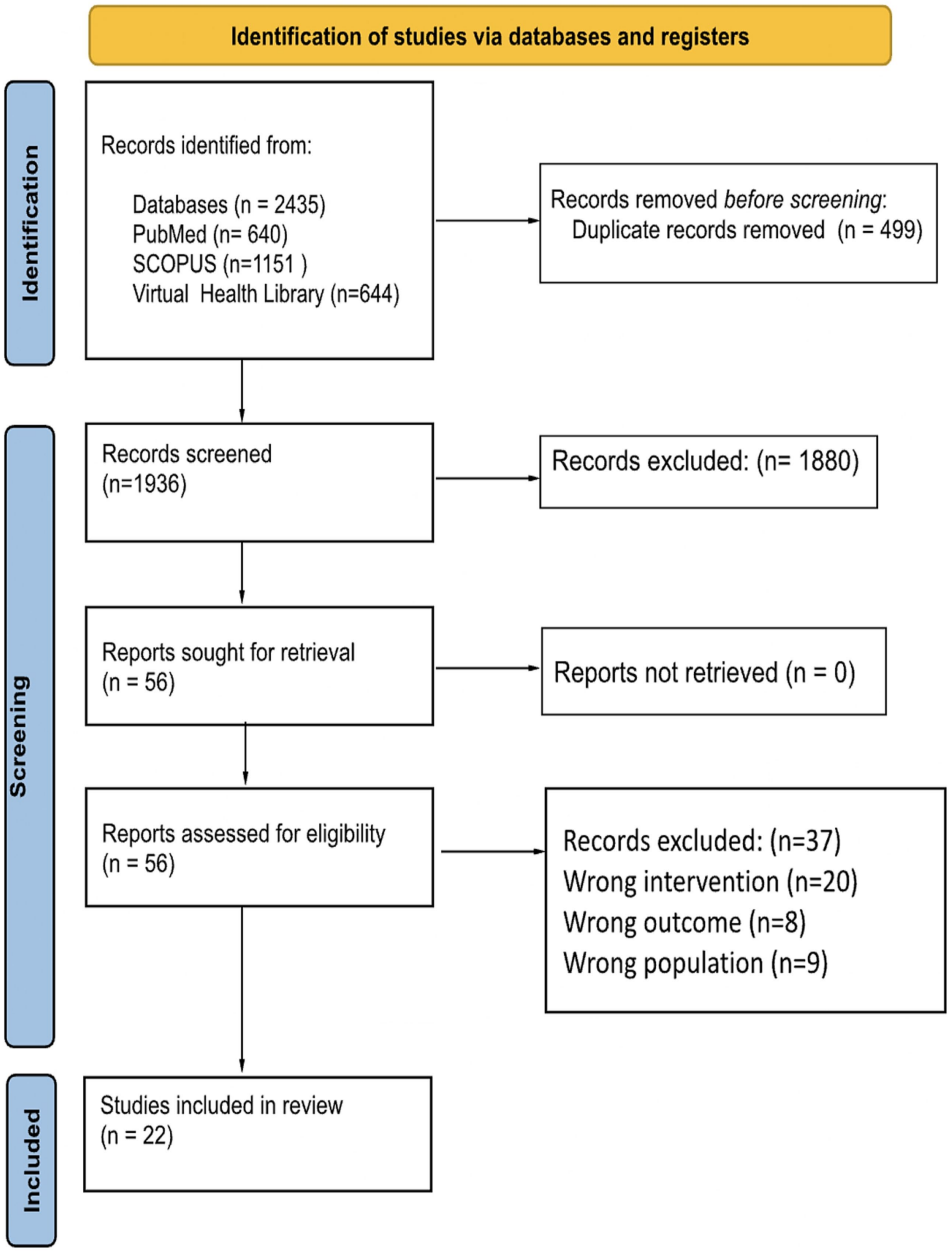
PRISMA 2020 flowchart illustrating the full selection process, including identification, screening, eligibility assessment, and final inclusion of studies in the systematic review; arrows indicate progression through each phase and boxes display the number of records included or excluded at each stage.

We searched PubMed, Scopus, and the Virtual Health Library from inception until February 10, 2024, yielding a total of 2,435 articles; of these, 499 were duplicates, leaving a total of 1936 articles for eligibility through title, abstract, and keyword analysis by two independent and blinded reviewers. We finally included 22 articles, 21 animal models study (7, 21–40) and one human (41); the complete selection process is presented in Figure 1.

### Risk of bias

Risk of bias was assessed with the SYRCLE tool for animal studies (Table 1) and with the NHLBI Quality Assessment Tool for the human study (Table 2). The human study (41) had 10 out of 14 questions answered as “yes,” representing 71.4% of affirmative responses and, therefore, a moderate risk of bias. All animal studies lacked reporting of complete information regarding domains related to random sequence generation, allocation concealment, random housing reporting, performance blinding, random outcome assessment, detection blinding, and selective reporting.

**Table 1:** Risk of bias assessment with the SYRCLE tool.

Study ID	Author	Domain 1	Domain 2	Domain 3	Domain 4	Domain 5	Domain 6	Domain 7	Domain 8	Domain 9	Domain 10
1	Vecchia et al.	Unclear	Low	Unclear	Unclear	Unclear	Unclear	Unclear	Low	Unclear	Low
2	Tang et al.	Unclear	Low	Unclear	Unclear	Unclear	Unclear	Unclear	Low	Unclear	Low
3	Zhang et al.	Unclear	Low	Unclear	Unclear	Unclear	Unclear	Unclear	Low	Unclear	Low
4	Niquet et al.	Unclear	Low	Unclear	Unclear	Unclear	Unclear	Unclear	Low	Unclear	Low
5	Xiong et al.	Unclear	Low	Unclear	Unclear	Unclear	Unclear	Unclear	Low	Unclear	Low
6	Garcia et al.	Unclear	Low	Unclear	Unclear	Unclear	Unclear	Unclear	Low	Unclear	Low
7	Alqahtani et al.	Unclear	Low	Unclear	Unclear	Unclear	Unclear	Unclear	Low	Unclear	Low
8	Wang et al.	Unclear	Low	Unclear	Unclear	Unclear	Unclear	Unclear	Low	Unclear	Low
9	Vaillancourt et al.	Unclear	Low	Unclear	Unclear	Unclear	Unclear	Unclear	Low	Unclear	Low
10	Santi et al.	Unclear	Low	Unclear	Unclear	Unclear	Unclear	Unclear	Low	Unclear	Low
11	Statler et al.	Unclear	Low	Unclear	Unclear	Unclear	Unclear	Unclear	Low	Unclear	Low
12	Smith et al.	Unclear	Low	Unclear	Unclear	Unclear	Unclear	Unclear	Low	Unclear	Low
13	Stewart et al.	Unclear	Low	Unclear	Unclear	Unclear	Unclear	Unclear	Low	Unclear	Low
14	Fournier et al.	Unclear	Low	Unclear	Unclear	Unclear	Unclear	Unclear	Low	Unclear	Low
15	Mckay et al.	Unclear	Low	Unclear	Unclear	Unclear	Unclear	Unclear	Low	Unclear	Low
16	Mckay et al.	Unclear	Low	Unclear	Unclear	Unclear	Unclear	Unclear	Low	Unclear	Low
17	Fan et al.	Unclear	Low	Unclear	Unclear	Unclear	Unclear	Unclear	Low	Unclear	Low
18	Peters et al.	Unclear	Low	Unclear	Unclear	Unclear	Unclear	Unclear	Low	Unclear	Low
19	McGowan et al.	Unclear	Low	Unclear	Unclear	Unclear	Unclear	Unclear	Low	Unclear	Low
20	Wang et al.	Unclear	Low	Unclear	Unclear	Unclear	Unclear	Unclear	Low	Unclear	Low
21	Chekhonin	Unclear	Low	Unclear	Unclear	Unclear	Unclear	Unclear	Low	Unclear	Low

Domains in the SYRCLE tool are as follows: 1. Sequence generation; 2. Baseline characteristics; 3. Allocation concealment; 4. Random housing; 5. Performance blinding; 6. Random outcome assessment; 7. Detection blinding; 8. Incomplete outcome data; 9. Selective reporting; 10. Other bias.

**Table 2:** Risk of bias assessment with the NHLBI quality assessment tools for Murman et al. (41).

Question	Response
1. Was the study described as randomized?	Yes
2. Was the method of randomization adequate?	Unclear
3. Was the treatment allocation concealed?	Unclear
4. Were study participants and providers blinded to treatment group assignment?	Yes
5. Were the people assessing the outcomes blinded to the participants’ group assignments?	Yes
6. Were the groups similar at baseline on important characteristics?	Yes
7. Was the overall drop-out rate ≤20%?	Yes
8. Was the differential drop-out rate ≤15% between groups?	Yes
9. Was there high adherence to the intervention protocols?	Yes
10. Were other interventions avoided or similar in groups?	Yes
11. Were outcomes assessed using valid and reliable measures?	Yes
12. Was the sample size sufficient to detect a difference with ≥80% power?	No
13. Were outcomes or subgroup analyses prespecified?	Unclear
14. Were all randomized participants analyzed in the group to which they were originally assigned?	Yes

### Ketamine influence in organic neurocognitive disorders

Regarding the only included human study (41), ten patients with Huntington’s disease underwent a double-blind within-subject pharmacological challenge in which escalating intravenous ketamine infusions (0.10, 0.40, and 0.60 mg/kg/h) were administered on one testing day and compared with placebo infusions on a separate, identical testing day. Cognitive performance was assessed across several domains, including verbal memory (Buschke Selective Reminding Test), visual memory (Washington Square Picture Memory Test), verbal fluency, attention (Digit Span Forward and simple reaction time), and motor agility (finger tapping) 2 days after ketamine exposure Overall, ketamine showed a dose-dependent negative influence on cognition, with significant impairments in immediate verbal recall beginning at 0.40 mg/kg/h, progressive decline in verbal fluency at 0.40 and 0.60 mg/kg/h, and slowing of reaction time at the highest dose.

**Figure 2:**
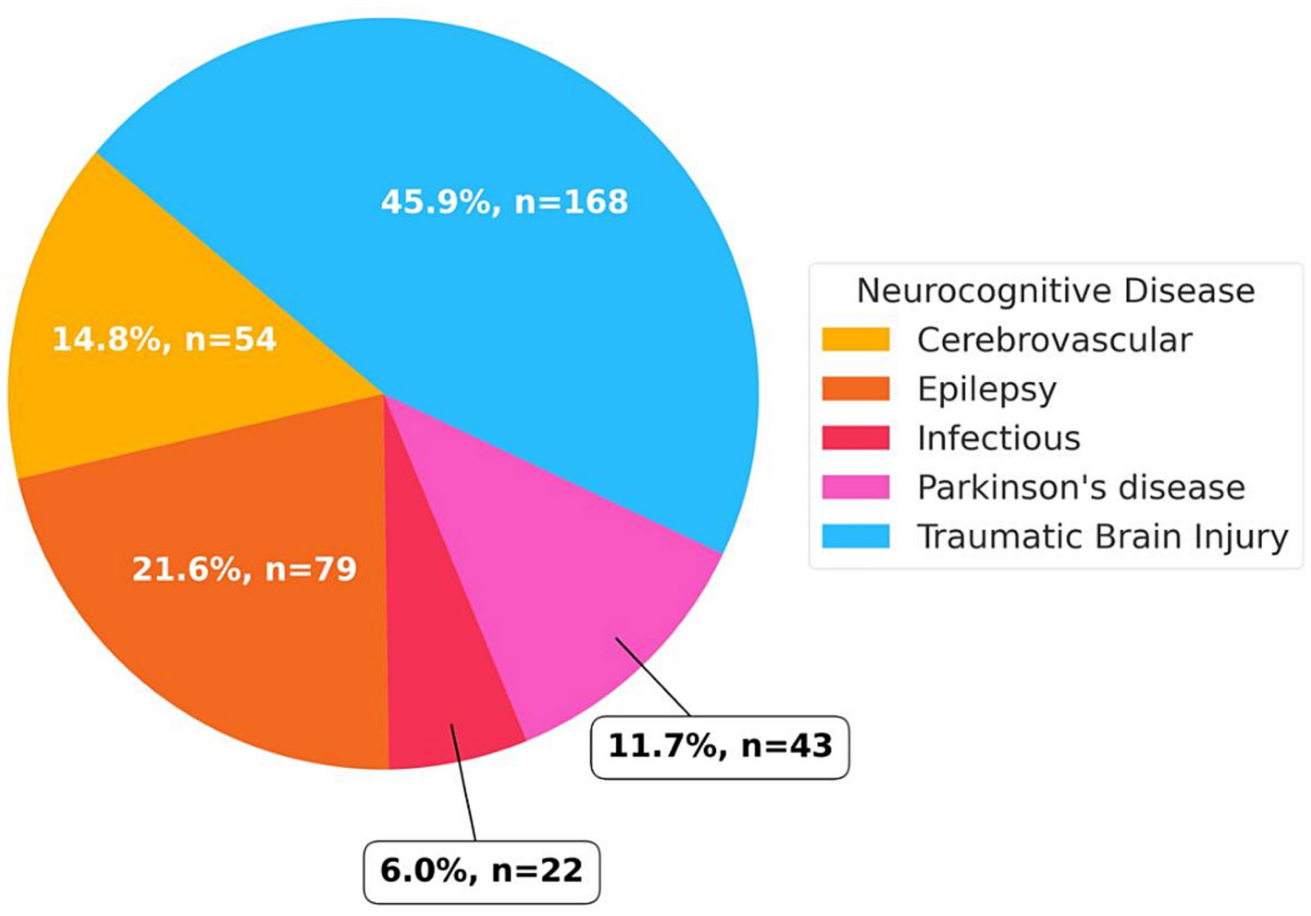
Distribution of organic neurological disease types among ketamine-treated subjects, showing absolute frequencies of rodents categorized by disease model; bars represent total subjects within each neurological condition, with colors differentiating disease groups such as traumatic brain injury, epilepsy, cerebrovascular disease, Parkinson’s disease, and infectious encephalopathy.

When looking at animal models of organic neurological disease with a neurocognitive impairment, we included a total of 366 rodents from 21 studies (7, 21–40); the relative and absolute frequencies of the type of neurological disease can be found in Figure 2. Table 3 showcases the most relevant characteristics of each study, including variables related with ketamine administration, disease model, and neurocognitive effect related to ketamine administration. In general, of the total rodents included 56.0% received ketamine <48 h after injury (*n* = 205) and 25.1% received it ≥48 h after injury (*n* = 92); in contrast 11.5% received it <48 h before injury (*n* = 42) and 27 rodents received ketamine both <48 h and ≥48 h after injury (7, 21–40). Most of the rodents received regular ketamine (53.3%), followed by its enantiomers S-ketamine in 17.7% and R-ketamine in 9.0%; 8.8% received both S- and R-ketamine, and 11.2% received a metabolite of ketamine—(2R,6R)-hydroxynorketamine (7, 21–40). Most of the models (*n* = 199) received a single dose of ketamine (54.5%) followed by multiple doses in 167 rodents; of these, the most common dosage was from 6 to 19 mg/kg in 44.3%, followed by ≥ 20 mg/kg in 42.3%, ≤ 5 mg/kg in 4.4%, and a combination of ≤ 5 mg/kg and 6 mg/kg–19 mg/kg in 9.0% (7, 21–40).

**Table 3:** Main characteristics of each study.

Author	Study design	Animal	Sex	N	Type of KET	Number of doses	KET dose	Route and time to test (after ketamine administration)	KET moment*	Neurocognitive disease	Cognitive domains evaluated	Influence in neurocognition
Niquet et al.	Experimental	Rats	Male	10	Ketamine	Multiple	(6 mg/kg–19 mg/kg)	Intraperitoneal; 7 days	Early Post Injury	Epilepsy	Spatial learning, Working memory	Negative
Alqahtani et al.	Experimental	Mice	Male	9	Ketamine	Single	(≥ 20 mg/kg)	Intraperitoneal; 3 days	Early Post Injury	Traumatic Brain Injury	Spatial learning, Working memory, Recognition memory, Short-term memory	Positive
Chekhonin et al.	Experimental	Rats	Male	20	Ketamine	Single	(≥ 20 mg/kg)	Intraperitoneal; 14 days, 1 month, and 2 months	Early Pre Injury	Cerebrovascular	Long-term memory, Aversive learning	Positive
Fan et al.	Experimental	Mice	Male	10	Ketamine	Single	(6 mg/kg–19 mg/kg)	Intraperitoneal; 2 hours	Early Post Injury	Parkinson's disease	Spatial learning, Working memory	Positive
Fournier et al.	Experimental	Rats	Male	4	Ketamine	Single	(≥ 20 mg/kg)	Subcutaneous; 14 days	Early Post Injury	Epilepsy	Associative learning, Emotional memory	Positive
Garcia et al.	Experimental	Rats	Male	41	(2R,6R)-hydroxynorketamine	Single	(≥ 20 mg/kg)	Intraperitoneal; 3 days	Delayed Post Injury	Traumatic Brain Injury	Recognition memory, Short-term memory	Positive
McGowan et al.	Experimental	Mice	Male	16	(R, S)-Ketamine	Single	(≥ 20 mg/kg)	Intraperitoneal; 30 days	Early Post Injury	Traumatic Brain Injury	Associative learning, Emotional memory, Depression-like behavior, Anxiety-like behavior	Positive
McKay et al.	Experimental	Rats	Male	8	Ketamine	Single	(≥ 20 mg/kg)	Subcutaneous; 90 days	Early Post Injury	Epilepsy	Spatial learning, Working memory, Associative learning, Emotional memory	Positive
McKay et al.	Experimental	Rats	Male	16	Ketamine	Single	(≥ 20 mg/kg)	Subcutaneous; 10 days	Early Post Injury	Epilepsy	Spatial learning, Working memory, Executive function, Cognitive flexibility	Positive
Peters et al.	Experimental	Mice	Both	27	Ketamine	Multiple	(6 mg/kg–19 mg/kg)	Subcutaneous; 1, 2, 7, 14, 21, 28 days	Both Post Injury	Traumatic Brain Injury	Spatial learning, Working memory, Cognitive flexibility	Positive
Smith et al.	Experimental	Rats	Male	9	Ketamine	Single	(6 mg/kg–19 mg/kg)	Intravenous; 2 days	Early Post Injury	Traumatic Brain Injury	Spatial learning, Working memory	Positive
Stewart et al.	Experimental	Rats	Male	15	Ketamine	Single	(≥ 20 mg/kg)	Subcutaneous; 30 days	Early Post Injury	Epilepsy	Associative learning, Aversion memory, Affective learning (emotional–pain processing)	Positive
Vaillancourt et al.	Experimental	Rats	Male	20	Ketamine	Single	(≥ 20 mg/kg)	Subcutaneous; 14 days	Early Post Injury	Epilepsy	Spatial learning, Working memory	Positive
Wang et al.	Experimental	Rats	Male	32	Ketamine	Multiple	(6 mg/kg–19 mg/kg)	Intraperitoneal; 2 hours, 1, 3, and 7 days	Early Post Injury	Traumatic Brain Injury	Spatial learning, Working memory, Cognitive flexibility	Positive
Xiong et al.	Experimental	Mice	Male	16	S-ketamine, R-ketamine	Single	(≤ 5mg/kg)	Intraperitoneal; 12 hours, 1, and 2 days	Early Post Injury	Cerebrovascular	Functional Impairment	Positive
Tang et al.	Experimental	Mice	Male	25	Esketamine	Multiple	(6 mg/kg–19 mg/kg)	Intraperitoneal; 1, 3 and 7 days	Early Post Injury	Traumatic Brain Injury	Functional Impairment	Positive
Vecchia et al.	Experimental	Rats	Male	33	Racemic Ketamine	Multiple	(≤ 5mg/kg)	Intraperitoneal; 1 day	Delayed Post Injury	Parkinson's disease	Social memory, social cognition, Exploratory behavior, Anxiety-like behavior	Positive
Wang et al.	Experimental	Mice	Male	22	Esketamine	Multiple	(6 mg/kg–19 mg/kg)	Intraperitoneal; 1 day	Early Pre Injury	Infectious	Spatial learning, Working memory, Cognitive flexibility	Positive
Zhang et al.	Experimental	Mice	Male	18	S-ketamine	Multiple	(6 mg/kg–19 mg/kg)	Intraperitoneal; 31 days	Delayed Post Injury	Cerebrovascular	Recognition memory, Short-term memory, social memory, social cognition, Spatial learning, Working memory	Positive
Santi et al.	Experimental	Rats	Male	6	Ketamine	Single	(6 mg/kg–19 mg/kg)	Subcutaneous; 44 days	Early Post Injury	Epilepsy	Spatial learning, Working memory	Unchanged
Statler et al.	Experimental	Rats	Male	9	Ketamine	Single	(≥ 20 mg/kg)	Intravenous; 20 days	Early Post Injury	Traumatic Brain Injury	Spatial learning, Working memory	Unchanged

KET, Ketamine; N, number of subjects that received ketamine. * Early denotes administration of ketamine <48 h and late denotes administration ≥48 h.

**Figure 3:**
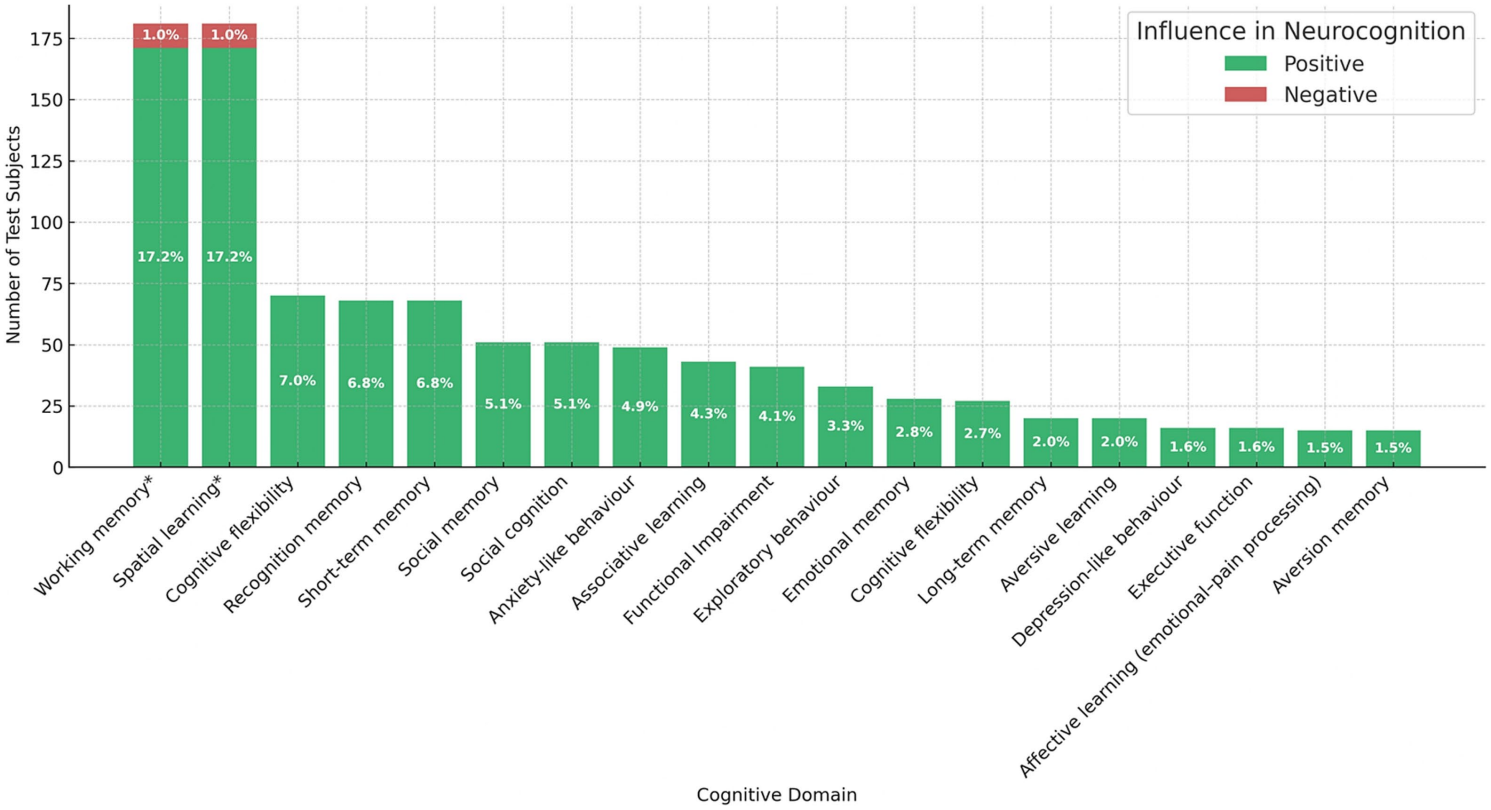
Effects of ketamine on cognitive domains by number of test subjects, showing the distribution of positive, negative, and unchanged outcomes across domains. Bars represent the absolute frequency of rodents evaluated in each cognitive domain. Color coding distinguishes the type of effect: green for positive outcomes, yellow for unchanged outcomes, and red for negative outcomes; working memory and spatial learning represent the highest concentration of positive effects.

Finally, when looking at the effect of ketamine on neurocognition, we detected an overall positive neurocognitive effect in 93.2% of subjects (*n* = 341), unchanged in 4.1% (*n* = 15), and negative in 2.7% (*n* = 10). Because subject-level summaries can disproportionately reflect studies with larger sample sizes and may not capture consistency across studies, results were also summarized at the study level. At this level, most animal studies reported overall positive cognitive effects, whereas a small number reported neutral or mixed findings, and none reported an overall detrimental cognitive effect. When stratifying these by type of cognitive domain affected (Figure 3), we discovered that working memory and spatial learning were the most affected domains with an improvement after ketamine administration in 17.2% of subjects (7, 21–28, 30–32, 35–40), and a negative effect in 1% of subjects (S10), for each.

**Figure 4:**
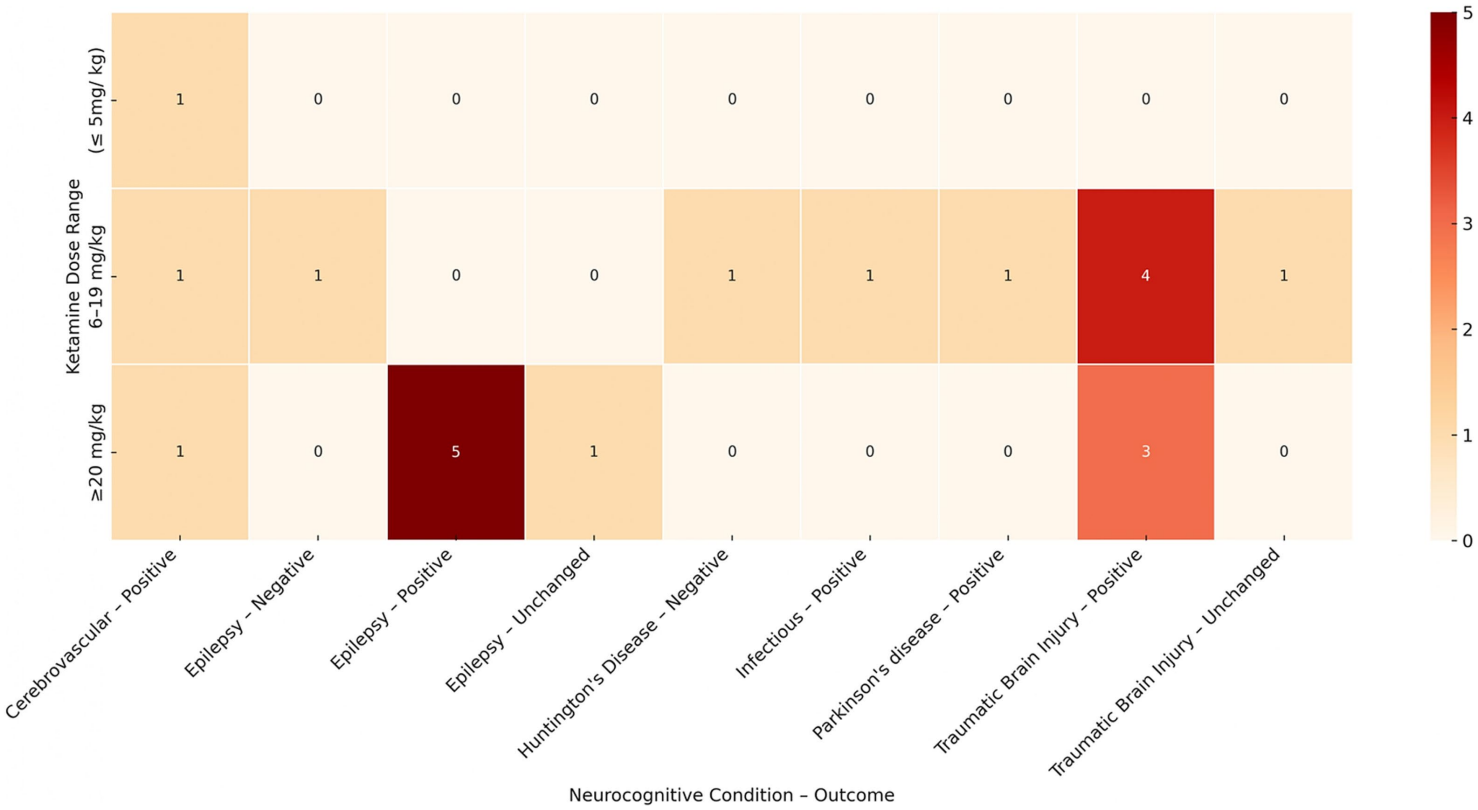
Heatmap of study density comparing ketamine dose range, neurological condition, and cognitive outcome. Colors indicate the number of studies reporting a positive, negative, or unchanged effect within each dose–disease combination, with darker shades representing a higher number of studies. Rows correspond to neurological conditions (e.g., traumatic brain injury, epilepsy, cerebrovascular disease), and columns correspond to ketamine dose ranges (<5 mg/kg, 6–19 mg/kg, ≥20 mg/kg).

Furthermore, these were the only two domains showing a negative effect (*n* = 10) as well as a null effect in only 15 subjects (33, 34). We also present a heatmap of study density (i.e., number of papers) comparing the general effect of ketamine (positive, negative, or unchanged) with the type of neurological organic disease and ketamine dose range in Figure 4. As can be seen, the majority of papers show a positive effect with ≥20 mg/kg of ketamine in epilepsy (*n* = 5 papers) and in traumatic brain injury (TBI) (*n* = 3 papers); subjects with TBI also show a positive influence with 6–19 mg/kg of ketamine (*n* = 4 papers) and subjects with cerebrovascular disease show a positive benefit regardless of the dosage. A negative effect was only reported in one study in epilepsy with 10 participants using a dose of 6 mg/kg–19 mg/ kg (29); whereas a null effect was found in two studies (33, 34), one in 6 epilepsy models receiving a dose of ≥ 20 mg/ kg, and one in 9 TBI models receiving a dose of 6 mg/kg–19 mg/ kg (Table 3).

## Discussion

The objective of this systematic review was to understand the impact of subanaesthetic dosing of ketamine on neurocognitive functioning in the context of organic neurocognitive disorders. In the field of neurocognitive rehabilitation, in which treatment may have limitations due neurodegenerative processes, impairments in awareness or other co-morbidities, the idea of having a neurotropic medication that may facilitate neurocognitive functioning is hopeful as it is imperative to entertain all possible avenues of enhancing functioning. Despite emerging research suggesting the possibility of beneficial effects on cognitive functioning, there is a paucity of research investigating the impact of subanaesthetic doses of ketamine on cognitive functioning in the context of adult population neurological disorder. While the current systematic review mostly focused on animal studies due to the content of extant literature regarding neurocognitive functioning and subanaesthetic doses of ketamine, the results we found indicate merit to investigating subanaesthetic doses in human neurological populations.

Across 22 studies, the evidence base is dominated by preclinical work (21 animal studies; n = 366 rodents) with wide variation in disease models, timing, and dosing, but a convergent signal of benefit: ketamine (most often racemic; typically 6–19 mg/kg or ≥20 mg/kg, given as single or multiple doses and frequently within 48 h of injury) was associated with improved neurocognition in ~93% of subjects, most consistently in working memory and spatial learning; null results were uncommon (~4%) and adverse cognitive effects rare (~3%). Patterns by indication and dose suggested particularly robust improvements in epilepsy and traumatic brain injury models -especially at ≥20 mg/kg-while cerebrovascular models showed benefits across dose ranges. Risk-of-bias reporting in animals was generally incomplete across key domains, limiting certainty about effect sizes and reproducibility.

By contrast, the single human study (10 patients with Huntington’s disease) found short-term, dose-dependent decrements 2 days after escalating IV ketamine infusions, with immediate verbal recall and verbal fluency most sensitive (and simple reaction time affected only at the highest dose) (41); overall methodological quality was moderate. Taken together, the preclinical literature suggest domain-specific cognitive improvements under certain dosing/timing conditions. In contrast the single human study identified in this review reported dose-dependent short-term cognitive impairments following ketamine administration. This discrepancy highlights a translational gap and underscores the need for rigorously designed human trials that align dosing, timing, and domain-specific cognitive outcomes with the most consistent preclinical findings, while carefully monitoring for transient cognitive adverse effects.

Emerging clinical work also suggests that response to esketamine may vary according to baseline cognitive-affective and psychological constructs, including mentalization capacity, cognitive rigidity, psychache, and suicidality, underscoring the importance of measuring such variables and pre-specifying moderator analyses in future trials rather than assuming uniform treatment effects (42). In parallel, real-world evidence emphasizes the relevance of monitoring safety outcomes such as suicidality and self-harm and considering sex or gender as potential effect modifiers when evaluating ketamine-based interventions (43).

Although emerging evidence suggests that subanesthetic ketamine may exert beneficial effects on cognitive processes via its neuroplasticity-enhancing mechanisms, there remains a significant paucity of research directly investigating its effects within adult neurological populations. Therefore, this review predominantly drew upon animal studies due to the limited availability of human clinical research in the brain injured population. Nevertheless, the findings underscore the theoretical and translational utility of further exploring subanesthetic ketamine as a cognitive adjunct in neurorehabilitation for patients with organic neurological disorders.

With respect to ketamine use in neurological populations, it is essential to distinguish between potentially neuropsychologically impairing acute effects occurring during intoxication, **s**ubacute effects that emerge after acute intoxication has resolved, and longer-term plasticity-related changes. This distinction has important implications for how ketamine might be integrated into rehabilitation settings, as well as for setting appropriate expectations regarding the timing and nature of potential pro-cognitive changes for both clinicians and patients.

Clinical evidence supports the use of subanesthetic ketamine in treatment-resistant depression (7, 8). These findings provide a clinical foundation for exploring whether similar neuroplastic mechanisms may be leveraged in neurological conditions characterized by cognitive dysfunction. Substantial clinical research has demonstrated the safety and antidepressant efficacy of subanesthetic ketamine in treatment-resistant depression (7, 9), though the manifestation and durability of positive cognitive effects remains less clearly established. There is no parallel precedent for symptom mitigation in the neurological population despite the overarching mechanism of action of neuroplasticity as well as some overlapping components of cognitive dysfunction in both categories of pathology. Moreover, psychiatric disorders often comorbid with neurological ones, as well as a bidirectional exacerbation of symptoms. More specifically, the relationship between neurocognitive functioning and psychiatric conditions is understood to be one of neurocognitive exacerbation in the context of psychiatric symptomology. It stands to reason, therefore, that in mitigating psychiatric symptomology might be an indirect way of viewing subanesthetic ketamine as a neurocognitive enhancer.

The main limitations of the current systematic review revolve around substantial heterogeneity across included studies, as well as the limited translational relevance of animal findings to human populations with brain injury. With regards to heterogeneity, studies varied significantly in key methodological parameters that are critical to understanding ketamine’s efficacy and applicability in humans. These included: the age and species of animals used, the type of ketamine, route of administration, exact dosing schedules, number of doses, timing between doses, time of neurocognitive assessment post-administration and the presence or absence of long-term follow-up.

Additionally, variability in injury models, pathologies examined, cognitive domains assessed, and post-injury assessment timelines further complicates the ability to synthesize results in a way that meaningfully informs clinical translation. These inconsistencies reduce the ability to draw definite conclusions about ketamine’s neurocognitive effects, especially when attempting to extrapolate to adult human populations with acquired brain injury. Moreover, inherent limitations in translating animal neurophysiology, behavior, and plasticity mechanisms to human systems must be acknowledged, particularly given the complexity of human cognition and psychological comorbidities. Finally, we could not preregister our protocol in PROSPERO given that our review included animal studies. However, no deviations occurred and the protocol was fulfilled in its entirety.

Subanesthetic ketamine, through its well-established effects on neuroplasticity, holds significant, yet largely unexplored potential as a cognitive enhancer, independent of its mood-stabilizing properties. The next frontier in ketamine research is within a neurology-based translational framework, wherein its pro-neuroplastic mechanisms can potentially be leveraged to support cognitive recovery and rehabilitation in non-psychiatric populations.

Clinicians treating neurocognitive disorders need to integrate conceptualization of neuropathology from a multi-disciplinary perspective. Neurocognitive disorders seen through the lens of psychiatry, neurology, and neurorehabilitation can thus be unified by the goal of optimizing cognitive function through plasticity-enhancing interventions. By integrating pharmacological agents like ketamine with behavioral and cognitive remediation strategies, we open the door to novel, interdisciplinary approaches to the treatment of organic neurological disorders, rooted not only in symptom mitigation, but potentially in cognitive restoration.

Future research should aim to establish safety and tolerability profiles for subanesthetic doses of ketamine in adults with acquired brain injury. Given the diversity of neurological conditions-ranging from traumatic brain injury to stroke, anoxic injury, and neuroinflammatory disorders, it will be essential to organize safety trials by neuropathology, injury severity, and time post-injury.

Once safety is established, the next critical step involves designing randomized controlled trials to assess the efficacy of subanesthetic ketamine. These trials should compare ketamine administration to natural trajectory of recovery, standard neurorehabilitation treatments, and combined ketamine integrated with neurorehabilitation protocols, to determine whether ketamine enhances responsiveness to neurorehabilitation interventions.

In addition, future studies should aim to establish standardized dosing protocols, including initial dosing following injury, timing of post-dosing assessments, optimal intervals between administrations, maintenance dosing schedules, and the timing of ketamine administration relative to neurorehabilitation interventions. In future trials, characterizing the temporal trajectory of ketamine’s neuropsychological effects will be essential, as acute administration may produce transient cognitive impairments that could subsequently evolve into longer-term, pro-cognitive neuroplastic changes outside the immediate intoxication window. This distinction is particularly important when integrating ketamine into neurorehabilitation, which should ideally be initiated during periods of heightened neuroplasticity. Longitudinal study designs with extended follow-up will also be critical to assess the durability of cognitive gains and to monitor for potential long-term adverse effects.

## Footnotes

1
https://www.crd.york.ac.uk/PROSPERO/help/eligibility

2
https://www.rayyan.ai/

3
https://www.nhlbi.nih.gov/health-topics/study-quality-assessment-tools
