# Supplementary material for: Ketamine as a potential cognitive enhancer in neurological disorders: evidence from preclinical and clinical studies
Source: Front Neurol. 2026 Mar 30;17:1786249. doi: 10.3389/fneur.2026.1786249 (PMC13070833; doi:10.3389/fneur.2026.1786249)
Supplement: Supplementary file 1 [file Table_1.DOCX]

**Supplementary Material 1**

**Search Criteria**

**PubMed:**

((Neurocognitive Impairment) OR ("Cognition Disorders"[Mesh]) OR ("Cognitive Dysfunction"[Mesh]) OR stroke OR (brain tumor) OR dementia OR (brain tumour) OR (organic neurocognitive impairment) OR (epilepsy) OR (traumatic brain disorder) OR encephalitis OR (systemic lupus erythematosus) OR (brain neoplasms) OR (brain cyst) OR (chiari malformation) OR (encephalopathies) OR (brain aneurysm) OR (brain arteriovenous malformation) OR (hydrocephalus) OR (Huntington’s Disease) OR (Parkinson’s Disease) OR (Alzheimer’s Disease) OR (traumatic brain Injury) OR TBI OR concussion OR (multiple sclerosis)) AND (Cognition OR (Cognitive function) OR (Cognitive functioning) OR Rehabilitation OR recovery OR (cognitive rehabilitation) OR (cognitive remediation) OR (measures of cognitive functioning) OR (self perception of cognitive functioning) OR Attention OR Memory OR Executive Function OR Processing Speed OR Language OR ("Cognition"[Mesh])) AND (ketamine OR esketamine OR arketamine OR ("Ketamine"[Mesh]) OR ("Esketamine" [Supplementary Concept]))

**Scopus:**

TITLE-ABS-KEY (((Neurocognitive Impairment) OR (stroke) OR (brain tumor) OR (dementia) OR (brain tumour) OR (organic neurocognitive impairment) OR (epilepsy) OR (traumatic brain disorder) OR (encephalitis) OR (systemic lupus erythematosus) OR (brain neoplasms) OR (brain cyst) OR (chiari malformation) OR (encephalopathies) OR (brain aneurysm) OR (brain arteriovenous malformation) OR (hydrocephalus) OR (Huntington’s Disease) OR (Parkinson’s Disease) OR (Alzheimer’s Disease) OR (traumatic brain Injury) OR (TBI) OR (concussion) OR (multiple sclerosis)) AND ((Cognition) OR (Cognitive function) OR (Cognitive functioning) OR (Rehabilitation) OR (recovery) OR (cognitive rehabilitation) OR (cognitive remediation) OR (measures of cognitive functioning) OR (self perception of cognitive functioning) OR (Attention) OR (Memory) OR (Executive Function) OR (Processing Speed) OR (Language)) AND ((ketamine) OR (esketamine) OR (arketamine)))

**BVS:**

((Neurocognitive Impairment) OR (alteración neurocognitiva) OR (stroke) OR (ictus) OR (brain tumor) OR (tumor cerebral) OR (dementia) OR (demencia) OR (brain tumour) OR (organic neurocognitive impairment) OR (alteración orgánica neurocognitiva) OR (epilepsy) OR (epilepsia) OR (traumatic brain disorder) OR (trastorno traumático cerebral) OR (encephalitis) OR (encefalitis) OR (systemic lupus erythematosus) OR (lupus eritematoso sistémico) OR (brain neoplasms) OR (neoplasias cerebrales) OR (brain cyst) OR (quiste cerebral) OR (chiari malformation) OR (malformación de Chiari) OR (encephalopathies) OR (encefalopatías) OR (brain aneurysm) OR (aneurisma cerebral) OR (brain arteriovenous malformation) OR (malformación arteriovenosa) OR (hydrocephalus) OR (hidrocefalia) OR (Huntington’s Disease) OR (enfermedad de Huntington) OR (Parkinson’s Disease) OR (enfermedad de Parkinson) OR (Alzheimer’s Disease) OR (enfermedad de Alzheimer) OR (traumatic brain Injury) OR (trauma craneoencefálico) OR (TBI) OR (concussion) OR (multiple sclerosis) OR (esclerosis múltiple)) AND ((Cognition) OR (cognición) OR (Cognitive function) OR (función cognitiva) OR (Cognitive functioning) OR (funcionamiento cognitivo) OR (Rehabilitation) OR (rehabilitación) OR (recovery) OR (recuperación) OR (cognitive rehabilitation) OR (rehabilitación cognitiva) OR (cognitive remediation) OR (remediación cognitiva) OR (measures of cognitive functioning) OR (medidas de funcionamiento cognitivo) OR (self perception of cognitive functioning) OR (autopercepción de funcionamiento cognitivo) OR (Attention) OR (Atención) OR (Memory) OR (Memoria) OR (Executive Function) OR (Función Ejecutiva) OR (Processing Speed) OR (Velocidad de Procesamiento) OR (Language) OR (Lenguaje)) AND ((ketamine) OR (Ketamina) OR (esketamine) OR (esketamina) OR (arketamine) OR (arketamina))
